# Endovenous laser ablation for saphenous veins and tributaries - the LEST technique

**DOI:** 10.1590/1677-5449.202201462

**Published:** 2024-09-03

**Authors:** Adriano Carvalho Guimaraes, Ricardo Herkenhoff Moreira, Petra Cristina Van Den Bogert, Sergio Quilici Belczak, Felipe Coelho, Walter Jr. Boim de Araujo

**Affiliations:** 1 V&P Hospital Dia, Santo Antônio da Platina, PR, Brasil.; 2 Hospital Nossa Senhora da Saúde, Santo Antônio da Platina, PR, Brasil.; 3 Clínica Circulação, Pato Branco, PR, Brasil.; 4 Centro Universitário São Camilo – CUSC, São Paulo, SP, Brasil.; 5 Pontifícia Universidade Católica do Paraná – PUCPR, Londrina, PR, Brasil.; 6 Universidade Federal do Paraná – UFPR, Hospital de Clínicas, Curitiba, PR, Brasil.

**Keywords:** varicose veins, thermoablation, venous insufficiency

## Abstract

Endovenous thermal ablation is now one of the most important techniques for treating chronic venous insufficiency. Technical refinements and technological innovations have made it possible to employ the method not only in the saphenous veins, but also to treat superficial veins such as varicose tributaries. We describe a technique for surgical treatment of varicose veins using endovenous laser thermal ablation employing multiple punctures and present the experience at our service with analysis of 601 cases operated using this technique. Thermoablative treatment of tributary veins with multiple puncture sites expands the applications for endolaser in treatment of lower limb varicose veins, providing, comprehensive, safe, and effective treatment.

## INTRODUCTION

Chronic venous disease (CVD) is an important public health problem and contributes to impaired patient quality of life. It causes sick leave and disability leave and demands long term clinical follow-up, overloading public and private health systems. It is estimated that 7 to 60% of the population have varicose veins and around 1 to 2% of the adult population have lower limb ulcers, 70 to 90% of which are attributable to venous reflux.^1^ Moreover, the mean incidence of CVD-related hospital admissions is 92 out of every 100,000 admissions.^2^

Surgical treatment should be prescribed whenever possible, and conventional surgery with ligation at the saphenofemoral junction (SFJ) and stripping of the saphenous vein is the most widely used technique. However, it is known that this technique is associated with a high relapse rate.^3^ In a cohort study^4^ with 5 to 8 years’ follow-up after surgical treatment (high ligation of the saphenous vein combined with phlebectomy), Ostler et al. found that 82% of the patients’ legs showed signs of relapse within the saphenous tract and 12% had reflux along the entire extent of the stripped great saphenous vein (GSV). They attributed the cause of relapse to neovascularization, which is identified histologically by presence of tortuous vessels with thin walls and absence of mural nerves.^5^

As described by Nyamekye et al.,^5^ tissue samples taken from the SFJ during re-exploration of the SFJ in patients with recurrent varicose veins were studied, finding neovascularization in 96.4% das samples. In 19 out of 28 samples, neovascularization was the only cause identified.

The advent of lasers in the 1960s enabled new possibilities for medical treatments and today laser thermal ablation is one of the most important techniques used to treat CVD.^6^ The main advantages are shorter recovery time, the possibility of office/outpatient treatment, reduced costs and social impact, less invasive procedures, and high resolution rates. These characteristics make it an excellent option for treatment of varicose veins, offering effective results with fewer hospital admissions.

Following certain safety recommendations, the endovenous thermoablation technique can also be used for superficial veins, such as varicose tributaries. Technological innovations, such as smaller caliber fibers and use of different wavelengths, supported by improved imaging diagnostics, have allowed rapid developments in this type of treatment.^7^

This study describes a technique for surgical treatment of varicose veins using endovenous laser thermal ablation and employing multiple punctures and presents the experience of one center with analysis of 601 cases operated using this technique.

## DESCRIPTION OF THE TECHNIQUE

After clinical consultation, physical examination, and color Doppler ultrasonography (CDU) examination for vein mapping and treatment planning, the disease and the available treatment options were explained to patients.

The endovenous laser thermoablation with multiple punctures technique was offered to patients who were considered eligible for the procedure. Those who agreed were asked to read and sign a free and informed consent form. The study was approved by the institutional Ethics Committee (No. 5.632.631).

**Figure 1:**
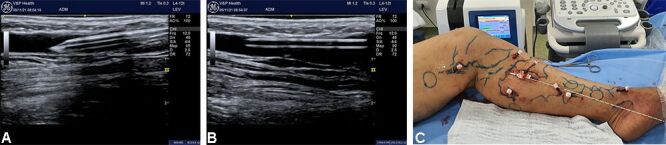
Images showing the multiple punctures made for treatment of tributaries and the great saphenous vein. (**A** and **B**) Ultrasound-guided placement of No. 16 IV catheter; (**C**) Appearance of the multiple punctures.
5 - Several different segments of the tributary veins, saphenous or otherwise, can be punctured at a distance of at least 7 to 10 mm from the skin (measured with ultrasound after perivenous infiltration of 0.9% saline);
6 - The patient is placed in the Trendelenburg position in order to provoke venous emptying;
7 - The 600 micra bare tip or radial laser fiber is inserted, adjusting the position of the fiber tip to 2 cm from the SFJ or saphenopopliteal junction (SPJ) (Figure 2);

The steps involved in the endovenous laser thermoablation with multiple punctures technique are as follows:

1 - Spinal anesthesia or sedation is given combined with tumescent local anesthesia;
2 - Venous access is obtained by ultrasound-guided puncture and a 6F introducer is advanced into the incompetent GSV;
3 - Punctures are made in tributary varicose veins with a 14 or 16 caliber Abbocath^®^ cannula, depending on the size of fiber that will be employed. A proximal torniquet can be used to facilitate punctures;
4 - After cannulation with the Abbocath,^®^ the needle is removed and a Luer-Lock^®^ stopper is fitted to prevent bleeding (Figure 1);

**Figure 2:**
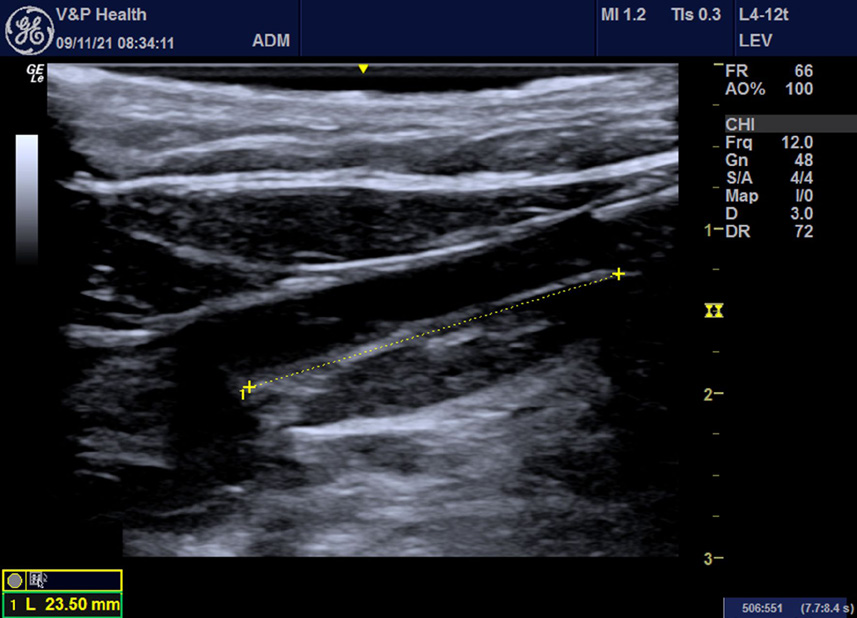
Longitudinal color Doppler ultrasound image in B-mode showing the distance from the fiber tip to the saphenofemoral junction.
8 - Venous tumescence is obtained using 0.9% saline, working in the foot-to-head direction, until reaching the SFJ (Figure 3);

**Figure 3:**
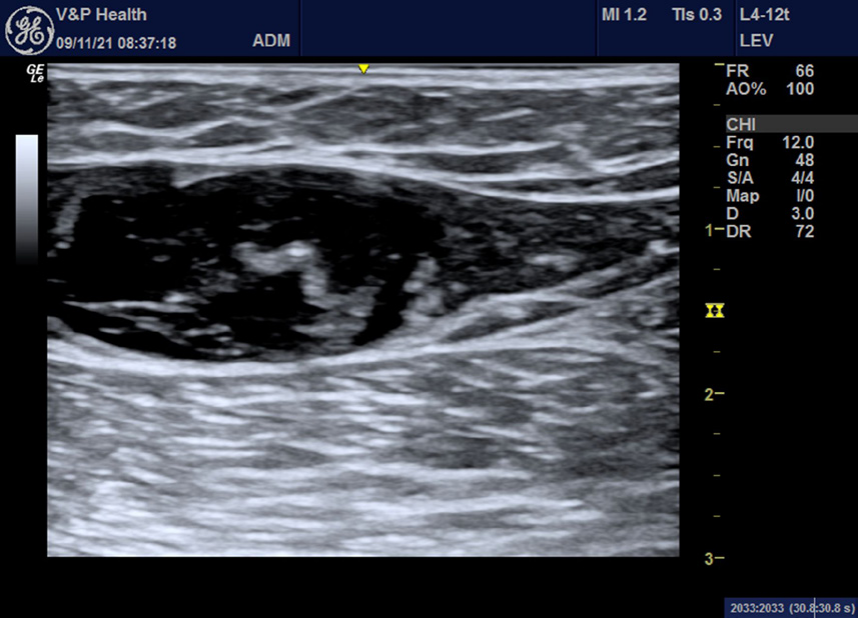
Color Doppler ultrasound image in B-mode showing the area of tumescence with the fiber central.
9 - Thermal ablation of the GSV is performed using a 1,470 nm wavelength laser, following a proximal-to-distal thermal ablation sequence and adhering to the following energy parameters, depending on the characteristics of the vein being treated (Figure 4):

**Figure 4:**
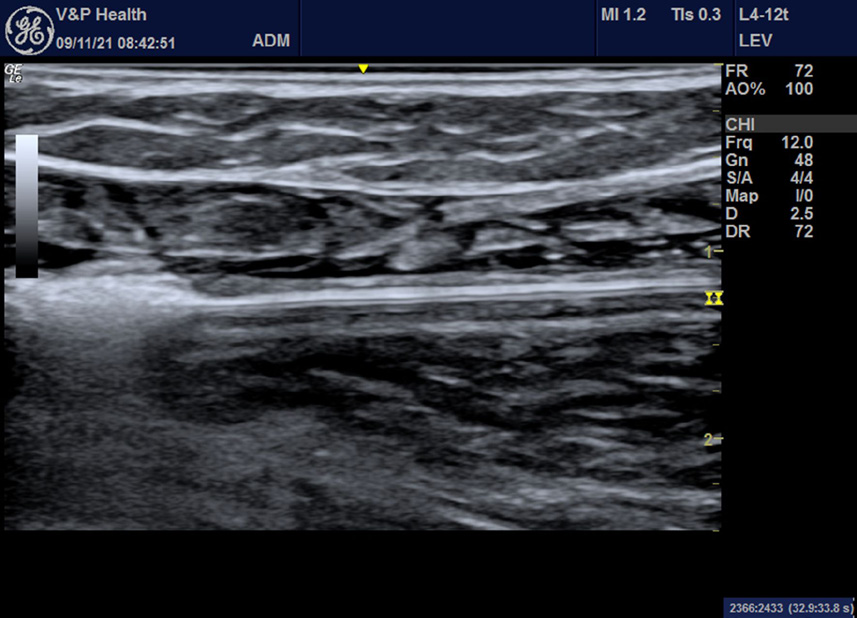
Color Doppler ultrasound image in B-mode showing the start of thermal ablation of the great saphenous vein.

**Figure 5:**
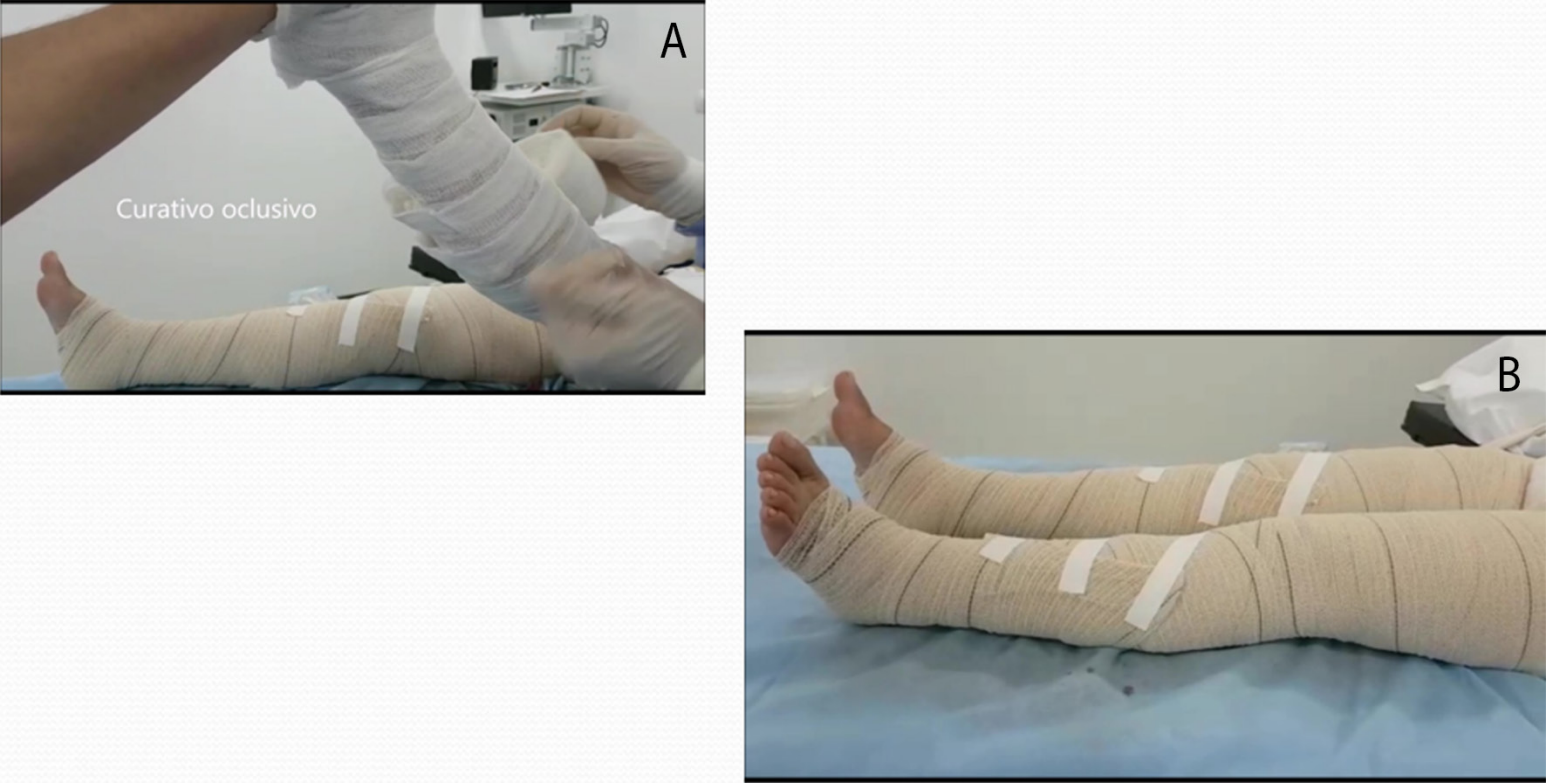
Compressive dressing and bandages, maintained for 24 h. (**A**) Starting occlusive dressing; (**B**) Dressing finished.
12 - The time spent in hospital is around 12 h;
13 - Patients are reassessed on the 7th, (Figure 6), 45th, and 90th days after the procedure.

- Power set at 9-12 W and linear endovenous energy density (LEED) of 80-100 J/cm, when using a straight fiber to treat the GSV in the thigh. When using a radial fiber, power is set to 5-8 W and the LEED used is 50-70 J/cm (with distance from junctions not less than 3 cm);
- Thermoablation of the GSV in the leg segment is achieved using LEED not exceeding 40 J/cm and at a distance from the skin of 7 to 10 mm;

10 - Once thermal ablation of the GSV is completed, proceed with treatment of the varicose tributaries. Using the accesses punctured in advance, a 600 micra bare tip laser fiber is inserted, followed by perivenous tumescence with 0.9% saline under ultrasound guidance. The laser fiber is kept in view using the transducer in the longitudinal plane. It is important to maintain the fiber static, since image quality is lost and the vein is compressed as tumescence is administered, making subsequent puncture difficult. The LEED employed for ablation of these veins is defined depending on their depth (before tumescence): LEED is 20-40 J/cm when less than 1 cm deep and 50-70 J/cm when deeper;
11 - Extrinsic compression is applied to the lower limb with gauze in rolls and sterile crepe bandages and maintained for 24 h (Figure 5);

**Figure 6:**
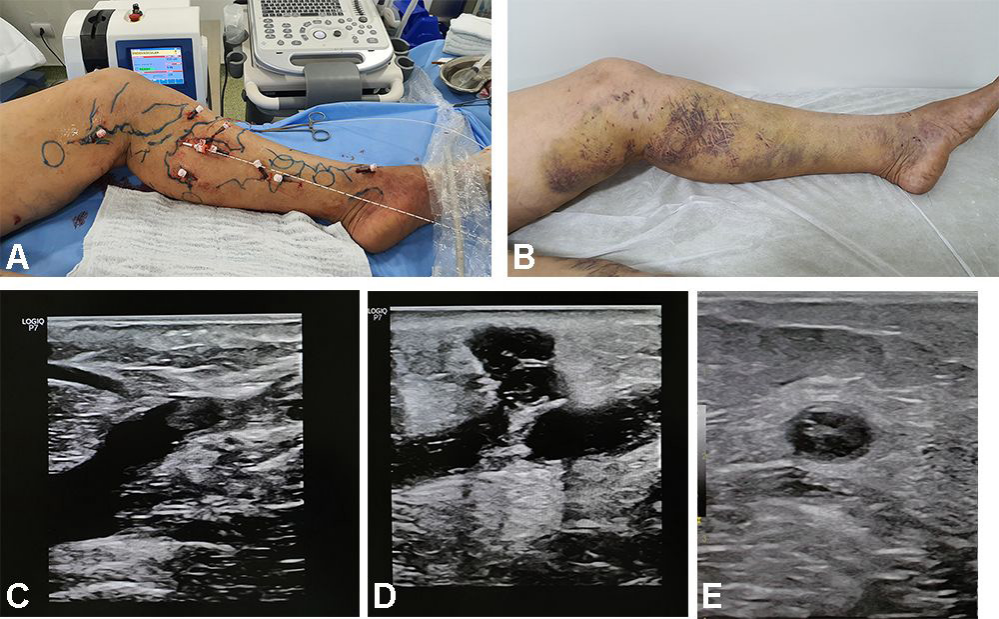
Ultrasound findings at 7 days. (**A**) Perioperative image showing the multiple punctures and catheters; (**B**) Postoperative appearance at 7 days; (**C**) Ultrasound image of the saphenofemoral junction close to the puncture site on the 7th postoperative day; (**D**) Ultrasound image of the great saphenous vein close to the puncture site on the 7th postoperative day; (**E**) Transverse view of the great saphenous vein in the thigh on the 7th postoperative day.

## DISCUSSION

**Figure 7:**
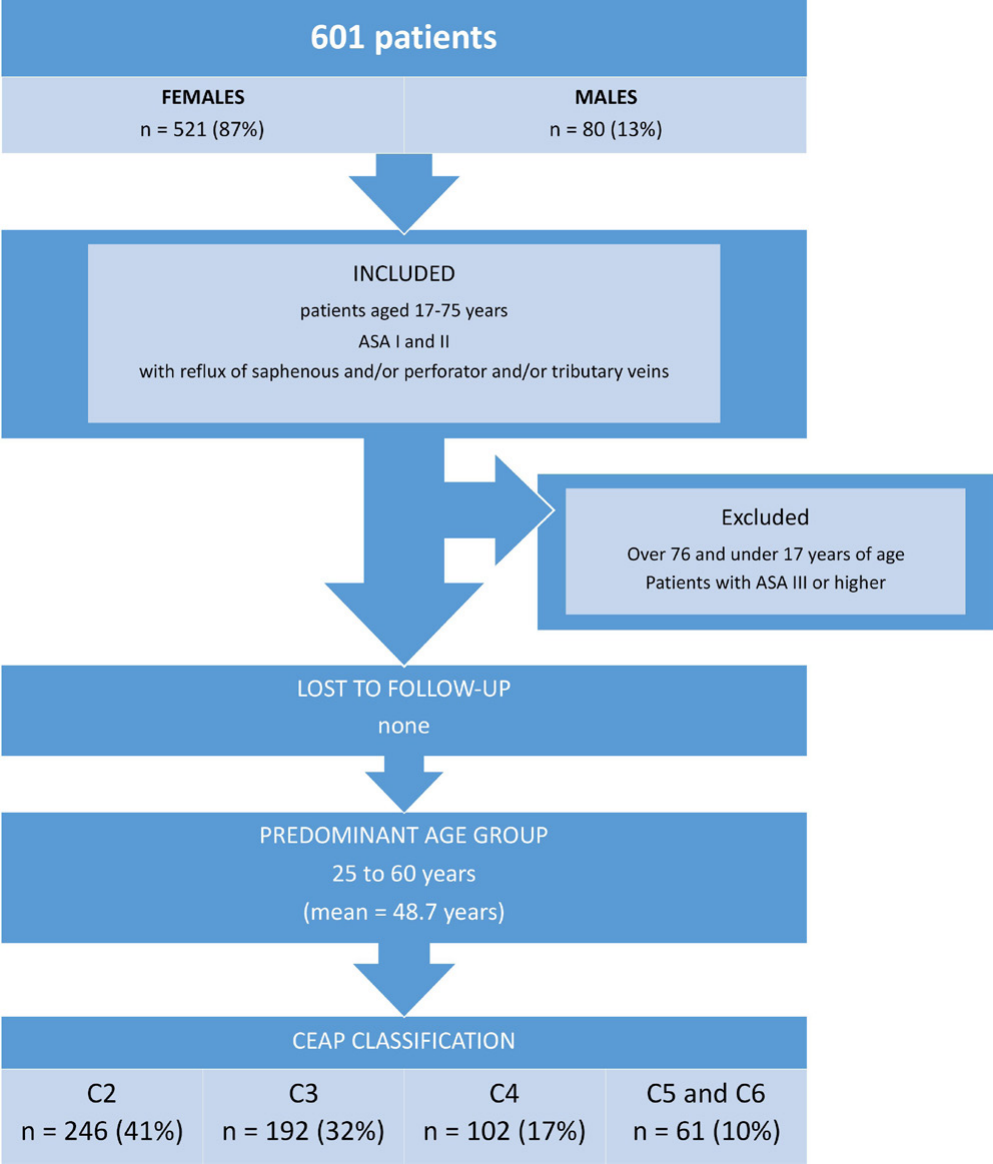
Flow diagram of patient selection. ASA = American Society of Anesthesiologists; CEAP = Clinical, Etiology, Anatomic and Pathophysiologic classification.

From January 2016 to March 2020, a total of 601 patients were treated using the technique, with ages ranging from 17 to 75 years, American Society of Anesthesiologists (ASA) surgical risk classes I and II, treated exclusively in a day hospital setting, with 521 (87%) females, and a predominant age group of 35 to 60 years (mean of 48.7 years). The majority of these patients had a Clinical classification, Etiology, Anatomic and Pathophysiologic (CEAP) classification of 2 or 3, breaking down as 246 (41%) C2, 192 (32%) C3, 102 (17%) C4, and the remaining 61 (10%) classified as C5 or C6. Patients were excluded if they had ASA surgical risk of III or greater or were aged under 17 or over 75 years. The patients selected had reflux of saphenous and/or perforating and/or deep tributary veins. None of these patients were lost to follow-up during the period (Figure 7). Unfortunately, the number of patients excluded was not recorded or analyzed, since these patients were not treated. This is why this ‘n’ is not shown in Figure.

The complications we observed (Table 1) were as follows:

**Table 1:** Complications observed (n = 601 cases).

**Complication**	**Number of complications**	**Percentage (%)**
Pulmonary embolism	1	0.17%
Deep venous thrombosis	7	1.17%
Arteriovenous fistula	1	0.17%
Hematoma/ecchymosis 7 days	601 (to greater or lesser degree)	100%
Paresthesias Up to 1 year	135	22.47%
Transitory hyperpigmentation Up to 1 year	217	36.10%
Infections/burns/deaths	0	0

- Seven cases of deep venous thrombosis, six of which were in muscular veins of the leg and one of which was in the popliteal vein.
- One case of pulmonary thromboembolism caused by type 2 endothermal heat induced thrombosis (EHIT) in the GSV, with no significant hemodynamic repercussions or sequelae.

Thromboembolic events were managed in rigorous accordance to current diagnostic protocols, including CDU of the lower limbs and chest angiotomography, and also in compliance with treatment guidelines, with precise administration of the appropriate dosage of rivaroxaban. It is important to point out that no signs or symptoms suggestive of postthrombotic syndrome were observed in any of these cases. The patient in the most critical case, involving popliteal vein thrombosis, has been in follow-up since 2019, with surveillance maintained to date. In the pulmonary thromboembolism case, meticulous follow-up covered a 21-month period, consolidating the temporal and comprehensive approach to monitoring of these specific cases.

- One hundred and thirty-five patients exhibited transitory paresthesia, in the majority self-limiting, without any need for specific treatment. Although the majority of patients who undergo varicose veins surgery enjoy excellent results and major morbidity and mortality are rare, minor morbidity, particularly cutaneous nerve injury, does remain a common problem. A review of the literature showed how much we do not know about this important complication and once again emphasized the need for further research. Sam et al.^8^ concluded that:
  - Cutaneous nerve injury can occur even with experienced surgeons and its presence is not therefore per se an indication of low quality care.
  - With the exception of CDU-guided foam sclerotherapy, none of the more recent minimally invasive techniques appear to offer significant protection against cutaneous nerve injury.
  - One patient developed a fistula from the anterior accessory saphenous vein to the superficial femoral artery, which was treated by ligation. This procedure was completed free from complications and the patient was reassessed on the 7th postoperative day, presenting free from complaints and with good healing of the surgical wound. Control CDU showed normal flow through the superficial femoral artery and the common femoral vein.^9^
  - Hematoma and/or ecchymosis were observed in 100% of cases at the 7-day follow-up consultation.
  - Cutaneous hyperpigmentation was observed in 217 cases, regressing gradually and completely within the first year.
  - There were no cases of infection, skin burns, or death.

The treatment principle of eliminating all possible sources of axial reflux and perforators was followed and all related varicose veins were also treated.

**Figure 8:**
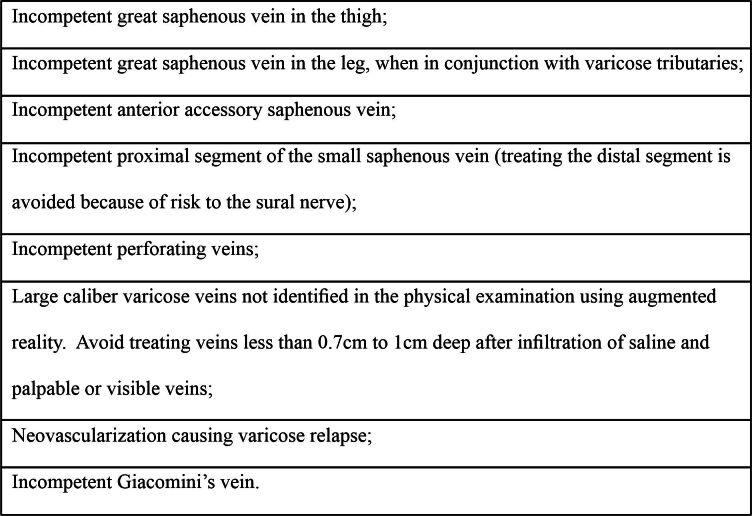
Indications for endovenous laser thermal ablation.

Figure 8 lists the main indications for endovenous laser thermal ablation with multiple punctures.

Axial reflux was found bilaterally in 270 (45%) cases in the sample and when each limb was assessed separately, the right lower limb was more affected (216; 36%).

The mean preoperative diameters of the incompetent GSVs treated were 6.6 mm at the SFJ and 5.6 mm at the thigh and leg and the mean preoperative diameter of the small saphenous vein was 3.5 mm.

Mean LEED used to treat the GSV was 90 J/cm when using the straight fiber to treat the GSV in the thigh. When using the radial fiber, power was set to 8 W and the LEED used was 50-70 J/cm (maintaining at least 3 cm distance to the junctions).

Thermal ablation of the GSV in the leg was conducted (assuming preoperative mapping indicated it was necessary) using LEED not exceeding 40 J/cm and at a distance from the skin of 7 to 10 mm. The mean number of punctures performed for treatment of tributaries was five (three to 15), while 15 was the maximum number of punctures in a single limb, and the mean LEED employed was 40 J/cm. The distance from the skin was maintained at 7 to 10 mm.

More superficial veins that were palpable on physical examination were treated with conventional phlebectomy. In contrast to our technique, the assisted total thermal ablation (ATTA) technique described by Amatuzi et al.^10^ involves ablation of all veins that can be punctured, regardless of their distance from the skin, using relevant variables to calculate the power to be used.

Treatment of superficial venous insufficiency with venous endolaser has been used for more than 15 years and has proven to be an excellent option in terms of the high rates of safety, efficacy, and patient satisfaction when compared with other surgical techniques. If we compare it to conventional surgery, venous endolaser is a less invasive technique that can achieve better esthetic results, while maintaining the efficacy of conventional stripping.^11^ Although the aim of this study was to describe the technique, it is impossible to ignore the observation that venous endolaser offers the benefits of minimally invasive surgery when compared to conventional surgery.

The technique of ablation with multiple punctures is intended to treat segments that would not be treated using the conventional phlebectomy technique, enabling treatment to be expanded. This is more obvious among obese patients and when dermatofibrosis or ulcers are present, but patients who are not part of these groups also benefit from the technique.

Use of endovenous laser in large caliber saphenous veins achieves good occlusion rates and this is not a contraindication to choosing the technique. Lasers with a wavelength of 1,560 nm have also proven effective for treatment of GSV with large diameters, as long as the LEED is adjusted to provide larger quantities of energy.^12,13^

The indications for use of endovenous laser have been extended beyond the treatment of axial saphenous reflux. Müller and Alm^14^ and Price et al.^15^ have described using endovenous laser to treat relapse of varicose veins originating at the SFJ by means of thermoablation via multiple punctures of segments of post saphenectomy neovascularization, which they described as “the hedgehog technique”.

Considering the variable characteristics of CVD presentations, a combination of endovenous laser and vein stripping makes it possible to treat large caliber GSVs in patients at advanced stages of disease, with good results over short-term follow-up.^16^

In line with proposals to extend the spectrum of veins that can be treated with endovenous laser, the possibility of using smaller caliber laser fibers enables management of incompetent perforating veins with satisfactory occlusion rates.^17^

Myers et al.^18^ described use of endovenous laser for concomitant treatment of the GSV and varicose tributaries. Tributaries were treatable with endovenous laser in 70% of cases, with treated segments measuring a mean of 14 cm in length, ranging from 3 to 38 cm.^18^

Our patient series has similarities to the results reported by Myers et al.,^18^ since the great majority of the tributary segments treated were less than 10 cm in length. This is because of the characteristic tortuosity of trunk varicose veins and the fiber’s poor “navigation” ability. This can be dealt with by making more punctures in order to completely ablate the entire varicose segment. Another aspect relevant to the success of treatment consists in identifying varicose tributaries that meet in the deepest segments of the subcutaneous or in areas with dermatofibrosis.

Systematic use of CDU is key to managing these tributaries, since it enables percutaneous access for ablation with the endovenous laser and therefore constitutes an extremely effective technique for treatment of varicose veins located in these deeper sites. We stress that this technique is not a substitute for conventional saphenectomy treatment or for surgical phlebectomies.

Nevertheless, we believe that when dealing with a disease with multiple clinical and anatomic presentations, the possibility of availing ourselves of additional options for surgical management of these patients can be of value to achieving better results.

We consider the technique described as a refinement, with the objectives of widening the scope for employment of endovenous laser in treatment of varicose veins, to minimize complications, and to improve results in patients who have the anatomic characteristics for which use of this technique is appropriate.

## CONCLUSIONS

Thermoablative treatment of tributary veins with multiple puncture sites expands the applications for use of endolaser to treat lower limb varicose veins. This technique is not a substitute for conventional treatment, but a refinement, with the objective of expanding treatment and minimizing complications, and may benefit patients to whom we were not previously able to offer adequate treatment of CVD, thus achieving comprehensive, safe, and effective treatment.
