# Supplementary material for: Endovenous laser ablation for saphenous veins and tributaries - the LEST technique
Source: J Vasc Bras. 2024 Sep 3;23:e20220146. doi: 10.1590/1677-5449.202201462 (PMC11404773; doi:10.1590/1677-5449.202201462)
Supplement: Translation

# Translation: Descrição da técnica LEST: *laser* endovenoso de safena e tributárias

**DOI:** 10.1590/1677-5449.202201461

**Authors:** Adriano Carvalho Guimaraes, Ricardo Herkenhoff Moreira, Petra Cristina Van Den Bogert, Sergio Quilici Belczak, Felipe Coelho, Walter Jr. Boim de Araujo

**Affiliations:** 1 V&P Hospital Dia, Santo Antônio da Platina, PR, Brasil.; 2 Hospital Nossa Senhora da Saúde, Santo Antônio da Platina, PR, Brasil.; 3 Clínica Circulação, Pato Branco, PR, Brasil.; 4 Centro Universitário São Camilo – CUSC, São Paulo, SP, Brasil.; 5 Pontifícia Universidade Católica do Paraná – PUCPR, Londrina, PR, Brasil.; 6 Universidade Federal do Paraná – UFPR, Hospital de Clínicas, Curitiba, PR, Brasil.

**Keywords:** varizes, termoablação, insuficiência venosa

## Abstract

Atualmente, a ablação térmica endovenosa é uma das principais técnicas para o tratamento da insuficiência venosa crônica. Com o refinamento técnico e as inovações tecnológicas, é possível aplicá-la, além de em veias safenas, em veias superficiais como as tributárias varicosas. Descrevemos uma técnica de tratamento cirúrgico de varizes por termoablação com *laser* endovenoso empregando-se múltiplas punções e a experiência do serviço após a análise de 601 casos operados com a técnica. O tratamento termoablativo de veias tributárias com múltiplos sítios de punção expande a aplicação do uso do endolaser no tratamento das varizes de membros inferiores de forma adequada, visando, assim, a um tratamento amplo, seguro e eficaz.

## INTRODUÇÃO

A doença venosa crônica (DVC) configura um importante problema de saúde populacional e contribui para piora da qualidade de vida de seus portadores. Ela ocasiona o afastamento das atividades laborais e exige seguimento clínico prolongado, sobrecarregando os sistemas de saúde públicos e suplementares. Estima-se que de 7 a 60% da população apresenta varizes e que cerca de 1 a 2% da população adulta apresenta úlcera de membros inferiores, das quais 70 a 90% são atribuídas a refluxo venoso^1^ . Além disso, a incidência média de internações hospitalares relacionadas à DVC é de 92 para cada 100.000 admissões^2^ .

O tratamento cirúrgico deve ser indicado sempre que possível, sendo a cirurgia convencional com ligadura da crossa e fleboextração de safenas a técnica mais utilizada. Entretanto, sabe-se que essa técnica se relaciona com uma alta taxa de recidiva^3^ . Ostler et al.^4^ identificaram, em um estudo de coorte com seguimento de 5 a 8 anos após tratamento cirúrgico (ligadura alta de safena associada a flebectomia), que 82% das pernas dos pacientes apresentavam sinais de recidiva dentro do compartimento safeno, sendo que 12% apresentavam refluxo em todo o território de veia safena magna (VSM) extirpada. Atribui-se como causa da recidiva a neovascularização, a qual se caracteriza histologicamente pela presença de vasos tortuosos, adelgaçamento de paredes e ausência de nervos murais^5^ .

Conforme publicado por Nyamekye et al.^5^ , foram estudadas amostras de tecidos da junção safenofemoral (JSF) ressecadas durante a reexploração da crossa de pacientes com veias varicosas recorrentes, sendo encontrada neovascularização em 96,4% das amostras. Em 19 de 28 amostras, a neovascularização foi a única causa identificada.

Com advento do *laser* na década de 60, surgiram novas possibilidades de tratamento na área médica, sendo hoje a ablação térmica umas das principais técnicas para o tratamento da DVC^6^ . Suas principais vantagens são período de recuperação mais curto, possibilidade de tratamento ambulatorial, redução de custos e impacto social, procedimento menos invasivo e alta resolutividade. Isso a tornou uma excelente escolha para o tratamento de veias varicosas, reunindo características que permitem um resultado efetivo com menos internações hospitalares.

Seguindo algumas recomendações de segurança, é possível aplicar a técnica de termoablação endovenosa em veias superficiais, como as tributárias varicosas. Inovações tecnológicas nos dispositivos como fibras de menor calibre e mudanças dos comprimentos de onda, aliadas à melhora no diagnóstico por imagem, fizeram com que houvesse rápida evolução desse tipo de tratamento^7^ .

O presente estudo se propôs a descrever a técnica de tratamento cirúrgico de varizes por termoablação com *laser* endovenoso empregando-se múltiplas punções e a experiência do serviço após a análise de 601 casos operados com a técnica.

## DESCRIÇÃO DA TÉCNICA

Após consulta clínica, exame físico e exame de eco-Doppler colorido (EDC) para mapeamento venoso e planejamento terapêutico, foram realizadas as explicações acerca da doença, bem como das opções terapêuticas disponíveis.

Foi oferecida a técnica de termoablação por *laser* endovenoso por meio de múltiplas punções para os pacientes que foram considerados elegíveis para o procedimento. Aqueles que concordaram foram orientados a assinar o termo de consentimento livre e esclarecido (TCLE). O estudo foi aprovado pelo Comitê de Ética da instituição (n.º 5.632.631).

O passo a passo da técnica da termoablação por *laser* endovenoso por meio de múltiplas punções consiste no seguinte:

1 - Raquianestesia ou sedação associada a anestesia local tumescente;
2 - Realização dos acessos venosos por meio de punção guiada por ultrassonografia e passagem de introdutor 6 F nas VSM insuficientes;
3 - Realização das punções com Abocath^®^ de calibre 14 ou 16 nas veias varicosas tributárias, a depender do perfil de fibra que será empregada. Para facilitar as punções, pode-se fazer o uso de um garroteamento proximal;
4 - Após a canulação com Abocath^®^, o mandril é retirado e uma tampa Luer-Lock^®^ é colocada para evitar sangramentos (Figura 1);

**Figura 1:** Imagens das múltiplas punções para tratamento das tributárias e da veia safena magna. (**A**) e (**B**) Passagem ecoguiada do cateter jelco n.º 16; (**C**) Aspecto das múltiplas punções.
5 - Diversos segmentos de veias tributárias safênicas ou não, com distância de pelo menos 7 a 10 mm da pele (medidas realizadas com ultrassom após a infiltração perivenosa com solução fisiológica a 0,9%), podem ser puncionados;
6 - O paciente é colocado em posição de Trendelenburg com o intuito de promover o esvaziamento venoso;
7 - A fibra de *laser bare tip* ou radial de 600 micras é introduzida, ajustando-se o posicionamento da ponta da fibra a 3 cm da JSF ou safenopoplítea (Figura 2);

**Figura 2:** Imagem com corte longitudinal de eco-Doppler colorido em modo B evidenciando a distância da extremidade da fibra à junção safenofemoral.
8 - É confeccionada a tumescência venosa com solução fisiológica a 0,9% no sentido podocefálico até a JSF (Figura 3);

**Figura 3:** Imagem de eco-Doppler colorido em modo B evidenciando a área de tumescência com a fibra central.
9 - A termoablação das VSM é realizada com aparelho de *laser* de comprimento de onda de 1.470 nm, seguindo a sequência da termoablação de proximal para distal, respeitando os seguintes parâmetros de energia, de acordo com as características da veia a ser tratada (Figura 4):

**Figura 4:** Imagem de eco-Doppler colorido em modo B demonstrando o início da termoablação da veia safena magna.

- Potência de 9-12 W e energia por centímetro linear de veia (*linear endovenous energy density* [LEED]) de 80-100 J/cm quando empregada a fibra reta para tratamento da VSM na coxa. Quando utilizada a fibra radial, ajusta-se a potência para 5-8 W e LEED entre 50-70 J/cm (distância das junções não menor que 3 cm);
- Termoablação da VSM em segmento de perna com LEED não maior que 40 J/cm e distância da pele de 7 a 10 mm;

10 - Finalizada a termoablação da VSM, segue-se o tratamento das tributárias varicosas. Por meio dos acessos previamente puncionados, é introduzida uma fibra de *laser bare tip* de 600 micras, seguido da tumescência perivenosa com soro fisiológico a 0,9% com o auxílio ultrassonográfico. A visualização da fibra de *laser* é mantida com o transdutor em plano longitudinal. É importante se atentar para a manutenção estática da fibra, já que, no momento da tumescência, ocorre perda da qualidade da imagem e compressão da veia, dificultando posteriores punções. A LEED utilizada para a ablação dessas veias é definida dependendo de sua profundidade (antes da tumescência): 20-40 J/cm quando menor que 1 cm e 50-70 J/cm quando maior;
11 - É realizada a compressão extrínseca do membro inferior com gaze em rolo e atadura de crepom estéril, mantida por 24 h (Figura 5);

**Figura 5:** Curativo compressivo com compressas e ataduras mantidos por 24 h. (**A**) Início do curativo oclusivo; (**B**) Curativo finalizado.
12 - O período de internação é de cerca de 12 h;
13 - Os pacientes são reavaliados no 7º (Figura 6), 45º e 90º dia após o procedimento.

**Figura 6:** Achados ultrassonográficos com 7 dias de evolução. (**A**) Imagem perioperatória demonstrando múltiplas punções e cateter; (**B**) Pós-operatório de 7 dias; (**C**) Aspecto ultrassonográfico da junção safenofemoral próximo ao ponto de punção com 7 dias de pós-operatório; (**D**) Aspecto ultrassonográfico da veia safena magna próximo ao ponto de punção com 7 dias de pós-operatório; (**E**) Corte transversal da veia safena magna em coxa com 7 dias de pós-operatório.

## DISCUSSÃO

Foram incluídos nessa técnica 601 pacientes no período entre janeiro de 2016 e março de 2020, com idades entre 17 e 75 anos, classificação no escore de risco cirúrgico da Sociedade Americana de Anestesiologistas (ASA) I e II, com tratamento exclusivamente em ambiente de hospital-dia, sendo 521 (87%) do sexo feminino e com faixa etária predominando entre 35 e 60 anos (média de 48,7 anos). A maioria dos pacientes tinha Classificação Clínica, Etiológica, Anatômica e Fisiopatológica (CEAP) 2 e 3, sendo 246 (41%) C2, 192 (32%) C3, 102 (17%) C4 e os 61 (10%) restantes eram classificados em C5 e C6. Foram excluídos pacientes com risco cirúrgico ASA III ou superior e com idade menor que 17 ou maior que 75 anos. Os pacientes selecionados tinham refluxo de safenas e/ou perfurantes e/ou tributárias profundas. Não houve perdas de seguimento desses pacientes no período (Figura 7). Infelizmente, não foram computadas as quantidades de excluídos, pois esses pacientes não foram operados. Por isso, esse ‘n’ não consta na Figura.

**Figura 7:** Fluxograma dos pacientes selecionados. ASA = Sociedade Americana de Anestesiologistas; CEAP = Classificação Clínica, Etiológica, Anatômica e Fisiopatológica.

Com relação às complicações, observamos (Tabela 1):

**Tabela 1:** Complicações observadas (n = 601 casos).

**Complicação**	**Número de complicações**	**Porcentagem (%)**
Embolia pulmonar	1	0,17%
Trombose venosa profunda	7	1,17%
Fístula arteriovenosa	1	0,17%
Hematoma/equimose 7 dias	601 (maior ou menor grau)	100%
Parestesias Até 1 ano	135	22,47%
Hiperpigmentação transitória Até 1 ano	217	36,10%
Infecção/queimadura/morte	0	0

- Sete casos de trombose venosa profunda, sendo seis casos em veias musculares de perna e um caso em veia poplítea.
- Um caso de tromboembolia pulmonar devido a trombose induzida pelo calor endovenoso (*endothermal heat induced thrombosis* [EHIT]) tipo 2 em VSM, sem repercussão hemodinâmica significativa ou sequela.

A conduta dos eventos tromboembólicos seguiu rigorosamente os atuais protocolos de diagnóstico, incluindo EDC de membros inferiores e angiotomografia de tórax, bem como as diretrizes terapêuticas recomendadas com a administração precisa da dose preconizada em bula de rivaroxabana. É importante ressaltar que, nesses casos, não foram observados sinais e sintomas evidentes de síndrome pós-trombótica. No cenário mais crítico, envolvendo a trombose de veia poplítea, o paciente permanece em acompanhamento desde 2019, persistindo a vigilância até o presente momento. Em relação à tromboembolia pulmonar, o seguimento meticuloso abrangeu um período de 21 meses, consolidando a abordagem temporal e abrangente no monitoramento desses casos específicos.

- Cento e trinta e cinco pacientes apresentaram parestesia transitória, na sua maioria autolimitada, sem necessidade de tratamento específico. Embora a maioria dos pacientes submetidos a cirurgia de varizes desfrute de um resultado excelente e morbimortalidade maior seja rara, a morbidade menor, particularmente a lesão do nervo cutâneo, continua sendo um problema comum. Uma revisão da literatura revelou o quanto não sabemos sobre essa importante complicação e enfatizou novamente a necessidade de mais pesquisas. Sam et al.^8^ concluíram que:
  - A lesão do nervo cutâneo pode ocorrer mesmo com cirurgiões experientes; sua presença não é, portanto, por si só um indicativo de cuidados de qualidade inferior.
  - Com exceção da escleroterapia com espuma guiada por EDC, nenhuma das técnicas mais recentes, minimamente invasivas, parecem oferecer proteção significativa contra lesão nervosa cutânea.
  - Um paciente evoluiu com fístula da veia safena acessória anterior para a artéria femoral superficial, sendo tratado por ligadura. O procedimento evoluiu sem intercorrências, e o paciente foi reavaliado no sétimo dia de pós-operatório, apresentando-se sem queixas e com boa evolução da ferida operatória. O EDC de controle evidenciou fluxo normal na artéria femoral superficial e na veia femoral comum^9^ .
  - Hematomas e/ou equimoses foram observados em 100% dos casos no retorno de 7 dias.
  - Hiperpigmentação cutânea aconteceu em 217 casos, regredindo completamente e gradualmente até o primeiro ano.
  - Não houve nenhum caso de infecção, queimadura de pele ou morte na casuística.

Respeitou-se o princípio terapêutico de abolir todas as fontes possíveis de refluxo axial, perfurantes, além do tratamento das varizes associadas.

As principais indicações da termoablação com *laser* endovenoso por meio de múltiplas punções estão listadas na Figura 8.

**Figura 8:** Indicações da termoablação com *laser* endovenoso.

O refluxo axial na amostra avaliada foi encontrado em 270 (45%) dos casos bilateralmente, e, quando se avaliou o membro isoladamente, o mais acometido foi o membro inferior direito (216; 36%).

O diâmetro médio pré-operatório das VSM incompetentes tratadas foi na crossa de 6,6 mm, na coxa 5,6 mm, perna e na safena parva de 3,5 mm.

A LEED média para tratamento da VSM foi de 90 J/cm, quando empregada a fibra reta para tratamento da VSM na coxa. Quando utilizada a fibra radial, ajustava-se a potência para 8 W e LEED entre 50-70 J/cm (distância das junções não menor que 3 cm).

A termoablação de VSM em segmento de perna foi feita, se mapeamento prévio indicava, com LEED não maior de 40 J/cm e distância da pele de 7 a 10 mm. Para o tratamento das tributárias, a média de punções realizadas por paciente foi de cinco (três a 15), sendo 15 a maior quantidade de punções em um único membro, e a LEED média empregada foi de 40 J/cm. A distância da pele se mantinha entre 7 e 10 mm.

Veias mais superficiais e palpáveis ao exame físico foram tratadas com flebectomia convencional. Diferindo da nossa técnica, a ablação térmica total assistida (ATTA) descrita por Amatuzi et al.^10^ realiza a ablação de todas as veias passíveis de punção, independentemente da distância da pele, utilizando variáveis implicadas para o estabelecimento do cálculo da potência.

O tratamento da insuficiência venosa superficial com endolaser venoso é utilizado há mais de 15 anos e tem se mostrado uma excelente opção pelos altos índices de segurança, eficácia e satisfação do paciente quando comparado a outras técnicas cirúrgicas. Se compararmos à cirurgia convencional, o endolaser venoso é uma técnica menos invasiva que pode alcançar melhores resultados estéticos, mantendo a eficácia da remoção convencional^11^ . Apesar de ser o intuito do trabalho a descrição da técnica, não foi possível descartar a observação de que o endolaser venoso fornece os benefícios da cirurgia minimamente invasiva se comparado à cirurgia convencional.

A técnica de ablação com múltiplas punções visa tratar segmentos que não seriam abordados na técnica de flebectomia convencional, possibilitando a ampliação do tratamento. Isso é mais evidente em pacientes obesos e quando há dermatofibrose ou úlcera, mas pacientes que não se encontram nesses grupos também se beneficiam da técnica.

O emprego do *laser* endovenoso em safenas de grande calibre apresenta boas taxas de oclusão, não constituindo uma contraindicação para a escolha da técnica. *Lasers* com comprimento de onda de 1.560 nm também se mostraram eficazes no tratamento de VSM de grandes diâmetros, desde que se proceda à adaptação da LEED para maiores quantidades de energia^12,13^ .

As indicações do uso do *laser* endovenoso têm sido expandidas para além do tratamento do refluxo axial de safenas. Müller e Alm^14^ e Price et al.^15^ descreveram o emprego do *laser* endovenoso para tratamento da recidiva de varizes oriundas da JSF por meio de termoablação via múltiplas punções nos segmentos de neovascularização pós-safenectomia, descrita como a “técnica do ouriço”.

Dadas as características variáveis da apresentação da DVC, a combinação das técnicas de *laser* endovenoso e fleboextração permite tratar VSM de grande calibre em pacientes nos estágios avançados da doença, com bons resultados no seguimento de curto prazo^16^ .

Em linha com a proposta de ampliação do espectro de veias passíveis de tratamento com *laser* endovenoso, a possibilidade de utilização de fibras de *laser* de menor perfil permite a abordagem de veias perfurantes incompetentes, com taxas satisfatórias de oclusão^17^ .

Myers et al.^18^ descreveram o uso do *laser* endovenoso para tratamento concomitante da VSM e das tributárias varicosas. As tributárias foram passíveis de tratamento com *laser* endovenoso em 70% dos casos, com segmentos tratados com 14 cm de extensão em média, variando de 3 a 38 cm^18^ .

Nossa casuística tem semelhança com os achados de Myers et al.^18^ , dado que, na grande maioria das tributárias tratadas em nossa série, os segmentos varicosos mediram menos de 10 cm. Isso pode ser explicado pela tortuosidade característica das varizes tronculares e pela falta de “navegabilidade” da fibra. Tal situação é contornada com a realização de mais punções para completar a ablação de todo o segmento varicoso. Outro aspecto relevante para o sucesso do tratamento consiste identificar tributárias varicosas que se encontram no segmento mais profundo do subcutâneo ou em áreas de dermatofibrose.

O uso sistemático do EDC é chave no manejo dessas tributárias, pois permite o acesso percutâneo para ablação com *laser* endovenoso e, assim, consiste em técnica extremamente efetiva para o tratamento das varizes localizadas nesses sítios mais profundos. Enfatizamos que essa técnica não é uma substituição ao tratamento convencional de safenectomia ou das flebectomias cirúrgicas.

Todavia, acreditamos que, ao lidar com uma doença com múltiplas apresentações clínicas e anatômicas, a possibilidade de lançarmos mão de alternativas para o manejo cirúrgico desses pacientes pode ser valiosa para alcançar melhores resultados.

Consideramos a técnica descrita como um aprimoramento, com os objetivos de ampliar a abrangência do emprego do *laser* endovenoso no tratamento de varizes, minimizar complicações e melhorar resultados em pacientes que apresentam as características anatômicas que contemplem o emprego da técnica.

## CONCLUSÃO

O tratamento termoablativo de veias tributárias com múltiplos sítios de punção expande a aplicação do uso do endolaser no tratamento das varizes de membros inferiores. A técnica não é uma substituição ao tratamento convencional, mas um aprimoramento, com o objetivo de ampliar o tratamento e minimizar complicações, podendo beneficiar pacientes para os quais antes não podíamos oferecer o tratamento da DVC de forma adequada, visando, assim, a um tratamento amplo, seguro e eficaz.
